# Smoking and epilepsy in national health interview survey: a cross-sectional study

**DOI:** 10.3389/fnhum.2025.1508526

**Published:** 2025-05-07

**Authors:** Xiaoping Huang, Qi Liu, Qingya Zhao, Qianqian Ji, Yiqiang Zhan

**Affiliations:** ^1^Department of Epidemiology, School of Public Health (Shenzhen), Sun Yat-sen University, Shenzhen, China; ^2^Institute of Environmental Medicine, Karolinska Institute, Stockholm, Sweden

**Keywords:** epilepsy, smoke, tobacco, NHIS, cross-sectional study

## Abstract

**Objective:**

This study aimed to examine the association between smoking and epilepsy in a nationwide cross-sectional analysis of US adults.

**Methods:**

Data from the National Health Interview Survey (NHIS) conducted between 2021 and 2022 were utilized, encompassing 57,088 participants aged 18–85 years with documented tobacco usage and self-reported epilepsy. Logistic regression models were employed to evaluate the relationship between smoking and epilepsy. The assessment of tobacco usage encompassed smoking status, cigarette smoking, pipe-filled tobacco smoking, and smokeless tobacco usage.

**Results:**

The overall prevalence of self-reported epilepsy in the sample was 1.76%, with smokers constituting 46.03% of cases. Individuals with epilepsy exhibited higher smoking rates compared to those without epilepsy. In the multivariate logistic regression model, smoking demonstrated a significant association with self-reported epilepsy (OR = 1.26, 95% CI: 1.07–1.47, *P* = 0.005). Subsequent stratified analyses showed consistent associations between smoking and self-reported epilepsy in all sex and ethnic subgroups.

**Conclusions:**

Our findings suggest that smoking is independently associated with self-reported epilepsy among US adults. Further investigation into the biological mechanisms underlying this association is warranted, contingent upon the validation of these results in an external population.

## Introduction

Epilepsy is a neurological non-communicable disease characterized by recurrent epileptic seizures, which manifest as repeated episodes of severe convulsive symptoms, varying in duration. Globally, there are more than 50 million people living with epilepsy, constituting 1% of the global disease burden, with nearly 80% of cases concentrated in low- and middle-income countries. In the United States, epilepsy has an annual economic impact of approximately 9.6 billion dollars (Yoon et al., 2009). Treatments for epilepsy are associated with diverse adverse cognitive and physical side effects, which may significantly impact the quality of life for individuals affected by the condition (Beghi and Giussani, 2019). Smoking represents a significant modifiable exposure among adults with epilepsy, with variations in its use among adults with epilepsy across different U.S. state (Dai and Richter, 2019). Smoking, recognized as the leading preventable cause of death and disability in the United States, accounts for approximately one in five deaths annually (Azagba et al., 2020). Notably, the prevalence of smoking among epilepsy patients worldwide exceeds that of the general population (Johnson et al., 2021; Terman et al., 2020; Zhen et al., 2023; Valodia, 2022; Cui et al., 2015; Ferguson et al., 2008; Hinnell et al., 2010; Torriani et al., 2016; Roberts, 2014; Roberts et al., 2015). These statistics underscore the critical need to elucidate the relationship between smoking and epilepsy.

While a 15-year study of 116,363 female nurses aged 25 to 42 years found that current smoking was associated with an increased risk of seizures (RR = 2.60) (Dworetzky et al., 2010), a retrospective cohort study following 394 patients between July 2017 and June 2019 revealed a 28% higher incidence of seizures among smokers compared to non-smokers (Raru et al., 2021). However, not all studies support the detrimental impact of smoking on epilepsy. For instance, a study showed no significant differences in various aspects between smokers with epilepsy and smokers without epilepsy (Johnson et al., 2021), and another study involving 278 male epilepsy patients from western China found that the smoking rate among epilepsy patients was lower than the overall smoking rate and suggested a beneficial effect of smoking on controlling epileptic seizures (Gao et al., 2017). The conflicting data from these studies may stem from differences in study designs and research methods. However, few studies have examined this association using recent, large-scale, and nationally representative data with detailed information on smoking behavior.

To further elucidate the relationship between smoking and epilepsy, we utilized recent NHIS data spanning 2021 and 2022, incorporating various styles of smoking to ascertain the precise association. Additionally, we conducted stratified analyses to investigate whether sex and ethnicity could modify this association.

## Methods

### Data source

The data utilized in this study were obtained from the National Health Interview Survey (NHIS), a long-standing data collection program of the National Center for Health Statistics (NCHS) continuously conducted since 1957 (https://www.cdc.gov/nchs/nhis/index.htm). The NHIS aims to gather comprehensive health data, providing precise and contemporary statistical insights into the prevalence, distribution, and impact of illness and disability in the United States, along with the corresponding services provided. The survey employs geographically clustered sampling techniques to select the sample of dwelling units for the NHIS. All participants or their legal guardians provided written informed consent.

### Study participants

A total of 57,133 adults participated in the NHIS surveys conducted in 2021 and 2022, following a content redesign that commenced in January 2019. Individuals with incomplete or missing self-reported epilepsy status information were excluded from our study. The final analysis included 57,088 participants.

### Epilepsy

The NHIS survey was administered through face-to-face interviews, and individuals who indicated that a doctor or health professional had diagnosed them with a seizure disorder or epilepsy were categorized as having epilepsy, without clinical diagnosis verification or medical record confirmation. Self-reported epilepsy has been recognized as a reliable tool for monitoring epilepsy within community settings. Studies have shown that the prevalence of self-reported epilepsy closely corresponds to figures obtained from clinical assessments of lifetime epilepsy and active epilepsy, supporting the validity of self-reported data in capturing the epidemiology of epilepsy (Brooks et al., 2012).

### Tobacco smoking

Following the World Health Organization's definition, a smoker is an individual who has smoked more than 100 cigarettes in their lifetime. Additionally, smoking status encompasses current smokers, former smokers, and never-smokers. Current smoker includes both daily and occasional smoking. To investigate the impact of different tobacco products on epilepsy, we gathered data on various types of tobacco used by participants, including cigars, pipes, and smokeless tobacco products.

Cigars refer to regular cigars, cigarillos, or filtered cigars, and do not include electronic cigars. Cigarillos are medium-sized cigars, sometimes sold individually or in packs of 5 or fewer, often with plastic or wooden tips. Filtered cigars resemble cigarettes and are typically brown. Similar to cigarettes, small filter cigars have sponge filters and are sold in packs of 20. Smoking pipes include regular pipes or water pipes, excluding electronic cigarettes and the use of pipes containing substances other than tobacco. Smokeless tobacco products encompass chewing tobacco, snuff, dip tobacco, or dissolvable tobacco but do not include nicotine replacement therapy products such as patches, gum, lozenges, or sprays, which are considered smoking cessation treatments.

### Potential confounders

We included additional covariates to mitigate the impact of confounding factors, comprising age, sex, ethnicity, body mass index (BMI), family poverty ratio, life satisfaction, and difficulty remembering/concentrating. Information on these covariates was obtained through interviews. Ethnicity was stratified into specific categories, including White only, Black/African American only, Asian only, American Indian or Alaska Native (AIAN) only, or AIAN combined with any other group. BMI was categorized as underweight, healthy weight, overweight, or obese. The family poverty ratio was calculated by comparing a family's income to the federal poverty threshold, which varies based on family size and composition and is expressed as a percentage. Values below 100% indicate that a family's income falls below the poverty threshold for their specific family size. Family poverty ratio was stratified into four categories: ≤ 1.1, 1.1~2.0, 2.0~4.2, and >4.2. Life satisfaction was assessed through inquiries regarding satisfaction levels, ranging from very satisfied to very dissatisfied. Participants self-reported the level of difficulty in remembering or concentrating, categorized as no difficulty, some difficulty, a lot of difficulty, or unable to do this at all.

### Statistical analyses

Continuous variables are presented as means ± standard deviation (SD), while categorical variables are expressed as frequencies and percentages. When comparing two independent groups, Student's *t*-test was employed for continuous variables, and Chi-square tests were used for categorical variables. All statistical analyses were conducted using appropriate complex survey procedures to account for the multistage, stratified sampling design of the NHIS, with sampling weights, strata, and primary sampling units to ensure national representativeness. Multivariable logistic regression models were utilized to assess the association between smoking and self-reported epilepsy, yielding an odds ratio (OR) with a 95% confidence interval (CI) as the effect measurement. Age, sex, ethnicity, BMI, family poverty ratio, life satisfaction, and difficulty remembering/concentrating were considered as additional confounding factors. A significance level of *P* < 0.05 was used to determine statistical significance, and all tests were two-sided. All statistical analyses were conducted using R 4.0 (R Core Team, Vienna, Austria).

## Results

### Characteristics of the study participants

Table 1 provides an overview of the baseline characteristics of the 57,088 study participants. The mean age at baseline was 48.12 ± 18.42 years. Men accounted for 48.48% of the participants, and individuals identifying as White comprised 76.84% of the participants. The prevalence of epilepsy was 1.76% (*N* = 1,007), with those of 1.67% among men (*N* = 417) and 1.98% among women (*N* = 590). Significant differences were observed in sex, race, BMI, family poverty ratio, life satisfaction, and difficulties with remembering or concentrating between individuals with epilepsy and those without (*P* < 0.05). Patients with epilepsy showed a higher prevalence of obesity (*P* < 0.001), reported lower life satisfaction (*P* < 0.001), and experienced challenges with cognitive function (*P* < 0.001). Furthermore, households with incomes below the federal poverty level had higher prevalence of epilepsy (*P* < 0.001).

**Table 1:** Characteristics of the participants at baseline according to self-reported epilepsy in NHIS, the US.

**Characteristics**		**Epilepsy (*N =* 1,007)**	**Non-Epilepsy (*N =* 56,081)**	**Total (*N =* 57,088)**	***p***
Age		48.62 ± 17.11	48.10 ± 18.45	48.12 ± 18.42	0.933
	Missing	1	146	147	
Sex					0.009
	Male	417 (44.23%)	25,955 (48.55%)	25,955 (48.48%)	
	Female	590 (55.77%)	31,128 (51.45%)	31,128 (51.52%)	
	Missing	0	5	5	
Race					< 0.001
	White only	767 (78.55%)	41,622 (76.80%)	42,389 (76.84%)	
	Black/African American only	138 (14.30%)	6,371 (13.10%)	6,509 (13.12%)	
	Asian only	14 (1.38%)	3,498 (6.58%)	3,512 (6.48%)	
	AIAN only	14 (1.54%)	490 (1.04%)	504 (1.05%)	
	AIAN and any other group	22 (2.50%)	458 (0.84%)	480 (0.87%)	
	Other single and multiple races	16 (1.73%)	777 (1.65%)	793 (1.65%)	
	Missing	36	2,865	1,417	
BMI					< 0.001
	Underweight	21 (1.87%)	874 (1.71%)	895 (1.72%)	
	Healthy weight	280 (28.21%)	17,347 (31.40%)	17,627 (31.34%)	
	Overweight	303 (29.27%)	18,888 (34.05%)	19,191 (33.96%)	
	Obese	385 (40.64%)	17,642 (32.84%)	18,027 (32.98%)	
	Missing	18	1,330	1,348	
Family poverty ratio		3.14 ± 2.66	4.23 ± 2.99	4.21 ± 2.98	< 0.001
	≤ 1.1	248 (21.07%)	6,413 (11.38%)	6,661 (11.55%)	
	1.1–2.0	209 (21.04%)	8,855 (15.99%)	9,064 (16.09%)	
	2.0–4.2	321 (34.51%)	17,552 (31.62%)	17,873 (31.68%)	
	>4.21	229 (23.39%)	23,261 (41.01%)	23,490 (40.68%)	
	Missing	0	0	0	
Life satisfaction/dissatisfaction					< 0.001
	Very satisfied	307 (31.99%)	25,567 (46.96%)	25,874 (46.67%)	
	Satisfied	542 (56.13%)	27,135 (48.70%)	27,677 (48.84%)	
	Dissatisfied	86 (7.61%)	2,065 (3.45%)	2,151 (3.53%)	
	Very dissatisfied	44 (4.27%)	558 (0.90%)	602 (0.96%)	
	Missing	28	756	784	
Difficulty remembering/concentrating					< 0.001
	No difficulty	498 (49.34%)	44,277 (80.16%)	44,775 (79.59%)	
	Some difficulty	361 (34.91%)	10,433 (17.49%)	10,794 (17.81%)	
	A lot of difficulty	140 (14.76%)	1,303 (2.28%)	1,443 (2.51%)	
	Cannot do at all	7 (0.99%)	46 (0.07%)	53 (0.09%)	
	Missing	1	22	23	
Have you smoked at least 100 cigarettes in your entire life					< 0.001
	Yes	475 (46.03%)	20,243 (34.02%)	20,718 (34.23%)	
	No	499 (53.97%)	34,282 (65.98%)	34,781 (65.77%)	
	Missing	33	1,556	1,589	
Cigarette smoking status					< 0.001
	Current smoker	205 (20.61%)	6,347 (11.40%)	6,552 (11.57%)	
	Former smoker	269 (25.39%)	13,884 (22.60%)	14,153 (22.65%)	
	Never smoker	499 (54.00%)	34,282 (66.00%)	34,781 (65.78%)	
	Missing	34	1,568	1,602	
Ever smoked a cigar					0.18
	Yes	306 (31.4%)	16,056 (30.93%)	16,362 (28.97%)	
	No	668 (68.6%)	38,477 (69.07%)	39,145 (71.03%)	
	Missing	33	1,548	1,581	
Ever smoked a pipe filled with tobacco					0.406
	Yes	151 (14.38%)	7,938 (14.07%)	8,089 (14.07%)	
	No	823 (85.62%)	46,601 (85.93%)	47,424 (85.93%)	
	Missing	33	1,542	1,575	
Ever used smokeless tobacco					0.047
	Yes	122 (13.27%)	5,748 (10.93%)	5,870 (10.97%)	
	No	853 (86.73%)	48,796 (89.07%)	49,649 (89.03%)	
	Missing	32	1,537	1,569	

AIAN, American Indian or Alaska Native.

### Association between smoking and epilepsy

To explore the relationship between smoking and epilepsy, four logistic regression models were employed. The results presented in Table 2 demonstrate significant associations between smoking and epilepsy. In the univariate logistic regression model, smoking exhibited a significant association with epilepsy (OR = 1.65, 95% CI: 1.42–1.92, *P* < 0.001). We observed significantly increased odds of epilepsy among both former (OR = 1.37, 95% CI: 1.16–1.63, *P* < 0.001) and current smokers (OR = 2.21, 95% CI: 1.80–2.72, *P* < 0.001). Further analyses with a multivariable logistic regression model were performed to account for potential confounders. Smoking was significantly associated with epilepsy (OR = 1.26, 95% CI: 1.07–1.47, *P* = 0.005), when evaluating smoking status, only current smoking was statistically related to epilepsy (OR = 1.45, 95% CI: 1.17–1.80, *P* < 0.001).

**Table 2:** Smoking in relation to self-reported epilepsy in NHIS, the US.

**Characteristics**		**Model 1**		**Model 2**		**Model 3**		**Model 4**	
		**p**	**OR(95%CI)**	**p**	**OR(95%CI)**	**p**	**OR(95%CI)**	**p**	**OR(95%CI)**
Have you smoked at least 100 cigarettes in your entire life	No								
	Yes	<0.001	1.65 (1.42–1.92)	<0.001	1.60 (1.37–1.87)	<0.001	1.44 (1.23–1.68)	0.005	1.26 (1.07–1.47)
Cigarette smoking status	Never smoker								
	Former smoker	<0.001	1.37 (1.16–1.63)	0.002	1.32 (1.10–1.58)	0.013	1.26 (1.05–1.51)	0.196	1.13 (0.94–1.35)
	Current smoker	<0.001	2.21 (1.80–2.72)	<0.001	2.09 (1.70–2.57)	<0.001	1.71 (1.39–2.10)	<0.001	1.45 (1.17–1.80)
Ever smoked a cigar	No								
	Yes	0.263	1.10 (0.93–1.29)	0.201	1.12 (0.94–1.35)	0.113	1.16 (0.97–1.39)	0.519	1.06 (0.89–1.27)
Ever smoked a pipe filled with tobacco	No								
	Yes	0.808	1.03 (0.83–1.26)	0.705	1.04 (0.84–1.30)	0.533	1.05 (0.84–1.31)	0.365	0.90 (0.72–1.13)
Ever used smokeless tobacco	No								
	Yes	0.06	1.25 (0.99–1.57)	0.048	1.30 (1.00–1.68)	0.064	1.28 (0.99–1.65)	0.23	1.17 (0.90–1.52)

Model 1 adjusted none. Model 2 adjusted for age, sex, and ethnicity. Model 3 adjusted for attained age, sex, ethnicity, body mass index and family poverty ratio. Model 4 adjusted for attained age, sex, ethnicity, body mass index, family poverty ratio, life satisfaction and difficulty remembering/concentration. OR, odds ratio; CI, confidence interval.

In both the univariate and multivariate logistic regression models, we did not observe significant variations in the association between tobacco smoking and epilepsy across different tobacco types, as illustrated in Table 2.

### Stratified analysis

The association of smoking with epilepsy was stronger in women (OR = 1.33, 95% CI: 1.08–1.65, *P* = 0.009) than in men (Table 3). A statistically significant association was observed among women who were current smokers, and the magnitudes of the associations with other tobacco products showed similar trends, albeit with wider confidence intervals. Additionally, the analysis by ethnicity revealed that consistent positive associations between smoking and epilepsy across all ethnic groups, with wider confidence intervals observed among Black/African Americans.

**Table 3:** Multivariable-adjusted odds ratios (ORs) and 95% confidence intervals (CIs) for smoking in relation to self-reported epilepsy by gender strata in NHIS, the US.

**Characteristics**				**Model 1**		**Model 2**		**Model 3**		**Model 4**	
				**p**	**OR(95%CI)**	**p**	**OR(95%CI)**	**p**	**OR(95%CI)**	**p**	**OR(95%CI)**
Have you smoked at least 100 cigarettes in your entire life			No								
	All		Yes	<0.001	1.65 (1.42–1.92)	<0.001	1.60 (1.37–1.87)	<0.001	1.44 (1.23–1.68)	0.005	1.26 (1.07–1.47)
	Sex										
		Male	Yes	<0.001	1.54 (1.23–1.93)	0.002	1.46 (1.14–1.85)	0.044	1.29 (1.01–1.65)	0.25	1.16 (0.90–1.49)
		Female	Yes	<0.001	1.85 (1.52–2.25)	<0.001	1.72 (1.40–2.12)	<0.001	1.55 (1.26–1.91)	0.009	1.33 (1.08–1.65)
Cigarette smoking status			Never smoker								
	All		Former smoker	<0.001	1.37 (1.16–1.63)	0.002	1.32 (1.10–1.58)	0.013	1.26 (1.05–1.51)	0.196	1.13 (0.94–1.35)
			Current smoker	<0.001	2.21 (1.80–2.72)	<0.001	2.09 (1.70–2.57)	<0.001	1.71 (1.39–2.10)	<0.001	1.45 (1.17–1.80)
	Sex										
		Male	Former smoker	0.032	1.32 (1.02–1.71)	0.113	1.25 (0.95–1.65)	0.198	1.20 (0.91–1.60)	0.493	1.10 (0.83–1.47)
			Current smoker	<0.001	1.99 (1.45–2.73)	<0.001	1.83 (1.33–2.50)	0.031	1.42 (1.03–1.96)	0.197	1.24 (0.89–1.73)
		Female	Former smoker	<0.001	1.49 (1.18–1.88)	0.002	1.36 (1.06–1.73)	0.046	1.29 (1.01–1.65)	0.322	1.14 (0.88–1.46)
			Current smoker	<0.001	2.53 (1.96–3.26)	<0.001	2.33 (1.79–3.02)	<0.001	1.94 (1.50–2.52)	<0.001	1.63 (1.24–1.46)
Ever smoked a cigar			No								
	All		Yes	0.263	1.10 (0.93–1.29)	0.201	1.12 (0.94–1.35)	0.113	1.16 (0.97–1.39)	0.519	1.06 (0.89–1.27)
	Sex										
		Male	Yes	0.941	1.01 (0.80–1.28)	0.696	0.95 (0.75–1.22)	0.932	1.01 (0.79–1.30)	0.916	0.99 (0.77–1.26)
		Female	Yes	0.001	1.47 (1.16–1.86)	0.008	1.39 (1.09–1.77)	0.010	1.38 (1.08–1.76)	0.189	1.19 (0.92–1.53)
Ever smoked a pipe filled with tobacco			No								
	All		Yes	0.808	1.03 (0.83–1.26)	0.705	1.04 (0.84–1.30)	0.533	1.05 (0.84–1.31)	0.365	0.90 (0.72–1.13)
	Sex										
		Male	Yes	0.692	0.95 (0.73–1.24)	0.444	0.90 (0.69–1.18)	0.476	0.91 (0.69–1.19)	0.163	0.82( 0.63–1.08)
		Female	Yes	0.071	1.34 (0.97–1.85)	0.093	1.32 (0.95–1.83)	0.083	1.34 (0.96–1.86)	0.767	1.05 (0.75–1.48)
Ever used smokeless tobacco			No								
	All		Yes	0.06	1.25 (0.99–1.57)	0.048	1.30 (1.00–1.68)	0.064	1.28 (0.99–1.65)	0.23	1.17 (0.90–1.52)
	Sex										
		Male	Yes	0.377	1.13 (0.86–1.50)	0.632	1.07 (0.80–1.44)	0.559	1.09 (0.81–1.46)	0.811	1.04 (0.77–1.40)
		Female	Yes	<0.001	2.60 (1.64–4.10)	<0.001	2.39 (1.51–3.78)	<0.001	2.18 (1.38–3.45)	0.024	1.75 (1.08–2.83)

Model 1 adjusted none. Model 2 adjusted for age, sex, and ethnicity. Model 3 adjusted for attained age, sex, ethnicity, body mass index and family poverty ratio. Model 4 adjusted for attained age, sex, ethnicity, body mass index, family poverty ratio, life satisfaction and difficulty remembering/concentration. OR, odds ratio; CI, confidence interval.

## Discussion

In this comprehensive study involving a large and diverse sample of 57,088 non-institutionalized US adults, our findings revealed a significant 26% increased odds of epilepsy associated with smoking. This association remained robust even after adjusting for several potential confounding variables, indicating an independent relationship between smoking and epilepsy in the general adult population. These findings underscore the importance of considering smoking habits as a potential risk factor for epilepsy and highlight the need for further research and public health initiatives aimed at addressing this association.

Our findings align with previous studies investigating the association between tobacco usage and epilepsy (Dworetzky et al., 2010; Raru et al., 2021; Johnson et al., 2019; Eid, 2021; Rui et al., 2022; Wang et al., 2021; Johnson et al., 2018; Venkatesh et al., 2021; Leu et al., 2020). Clinical data from 524 epilepsy patients indicated that smoking correlated with elevated seizure frequency (Rui et al., 2022). A study of 427 epilepsy patients with cognitive impairment illustrated an independent association between current smoking status and heightened epilepsy risk (Wang et al., 2021). In addition to epidemiological evidence, experimental animal models underscored the crucial role of tobacco components in inducing or aggravating epileptic seizures (Guntner et al., 2020; Laadraoui et al., 2018). However, the findings are not always consistent. The results of a study of 86 patients with epilepsy who had recently smoked showed that there were no differences between smokers with epilepsy and smokers without epilepsy in several aspects (Johnson et al., 2021). A study that included 278 male epilepsy patients from western China found that smoking has a beneficial effect on controlling epileptic seizures (Gao et al., 2017). Results from another study of 92 patients showed that smokers with epilepsy were more likely to report seizure control than those without epilepsy (Johnson et al., 2019). Studies from experimental animal models also showed that nicotine may improve seizure control in people with Autosomal Dominant Sleep Related Hypermotor Epilepsy (Becchetti et al., 2015). It is worth noting that some prior studies have reported inconsistent or even protective effects of smoking on epilepsy. These discrepancies may stem from differences in study populations, cultural factors influencing smoking behaviors, the classification of epilepsy subtypes, or methodological variations. Our study adds clarity to this area of research and underscores the need for further prospective studies to validate and extend these findings.

The interaction between smoking and sex yielded a statistically significant result, suggesting a potentially complex relationship between these factors and epilepsy. Previous research has indicated that hormonal influences may contribute to increased seizure activity in women, with progesterone recognized as an anticonvulsant hormone and estrogen potentially exerting proconvulsant effects. This hormonal interplay may contribute to the observed interaction between smoking and sex in relation to epilepsy (Bangar et al., 2016). Furthermore, our study identified a higher prevalence of epilepsy and lower smoking rates among women from lower socioeconomic backgrounds. This finding may be indicative of a poorer prognosis for female patients with epilepsy (McHugh and Delanty, 2008). Additionally, the observed association between smoking and epilepsy among individuals of different racial and ethnic backgrounds raises important considerations. Disparities in healthcare quality, cultural beliefs, and other confounding factors intertwined with self-reported epilepsy and race may underpin the amplified risk of epilepsy among individuals from various racial and ethnic groups. It is plausible that diverse racial or ethnic preferences impact self-identification with clinical terminologies like epilepsy among minority populations and immigrants, potentially influencing their responses to surveys on epilepsy. Notably, the confidence intervals for some racial and ethnic subgroups, particularly Black/African American participants, were wide, reflecting limited statistical power due to smaller sample sizes. Further large-scale, prospective studies specifically targeting underrepresented populations are warranted to validate these preliminary observations and clarify the complex interplay of smoking, race/ethnicity, sex, and socioeconomic factors in relation to epilepsy (Sirven et al., 2005; Kroner et al., 2013).

Numerous potential biological mechanisms have been proposed to elucidate the connection between smoking and epilepsy. One significant link is the association of autosomal dominant nocturnal frontal lobe epilepsy (ADNFLE) with mutations in the gene encoding the nicotinic acetylcholine receptor (nAChR) subunit, highlighting the involvement of the nicotinic system in epilepsy. Additionally, smoking is recognized to induce cerebrovascular atherosclerosis, leading to neuronal damage and dysfunction within the neural electrical network, which may contribute to the development of epileptic seizures (Sen et al., 2018). Moreover, smoking has been associated with an increased risk of stroke, which in turn can be a risk factor for seizures (Rui et al., 2022). However, the cross-sectional design of this study precludes any inference of temporal or causal relationships between smoking and epilepsy. An alternative hypothesis posits that individuals with epilepsy may be more inclined to smoke due to higher levels of psychological distress compared to the general population (Gorenflo et al., 2022; Im et al., 2016). Moreover, studies have shown a strong link between smoking and a lower risk of Parkinson's disease, potentially attributable to the neuroprotective properties of nicotine or other tobacco constituents. This well-documented relationship raises the intriguing possibility that individuals with epilepsy might similarly smoke as a form of self-medication to mitigate symptoms, warranting further investigation (Rose et al., 2024). These potential biological mechanisms underscore the intricate interplay between smoking and epilepsy, suggesting that both smoking behavior and epilepsy may influence each other through complex biological pathways.

This study possesses several notable strengths that enhance the robustness and relevance of its findings. Firstly, it draws upon data from the NHIS, a well-established and rigorously conducted survey with a substantial sample size, which lends credibility and statistical power to the study's results. Secondly, the use of a representative sample of non-institutionalized adult residents in the US enhances the generalizability of the findings, allowing for broader implications and applicability to the wider population. Thirdly, by categorizing subgroups within a moderately sized adult sample, the study avoids obscuring potential differences within these populations, providing a more nuanced understanding of the associations under investigation. However, it is important to acknowledge the limitations of this study. Firstly, the reliance on self-reported epilepsy data without clinical validation through biochemical assessments may introduce potential inaccuracies and biases. For example, although prior studies have validated the use of self-reported epilepsy data while non-differential misclassification would likely bias the observed association toward the null, potentially underestimating the true relationship between smoking and epilepsy. Secondly, the declining response rate of the NHIS survey raises concerns about increasing non-response bias, which could impact the representativeness of the study sample. Thirdly, the study's exclusive focus on cigarettes and other tobacco products highlights the need for further exploration of any potential links between epilepsy and e-cigarette use, given the increasing prevalence of e-cigarette use in recent years. Additionally, beyond the selected confounding variables addressed in this study, the relationship between epilepsy and smoking may be influenced by a range of other unavailable factors, such as genetic predisposition, comorbid psychiatric conditions, other substance use, cardiovascular disease, brain disease, and health insurance coverage in the US census region, emphasizing the need for comprehensive consideration of these additional factors in future research on this topic. These limitations underscore the complexity of the relationship between smoking and epilepsy and highlight areas for further investigation and refinement in future studies (Sapkota et al., 2020).

## Conclusion

In summary, our study found a significant association between smoking and an elevated prevalence of epilepsy in a representative sample of US adults. These findings hold important implications for both smoking prevention and epilepsy treatment efforts in the future. Specifically, our results suggest that targeted smoking cessation interventions may be particularly beneficial for individuals with epilepsy, with special attention to women and racial/ethnic minority groups, who demonstrated stronger associations in our analysis. Furthermore, such replication could have far-reaching implications for optimizing smoking prevention strategies specifically tailored to the epilepsy population. Additionally, further research into the mechanisms underlying the link between smoking and epilepsy is warranted to inform targeted interventions and improve the overall management of epilepsy in individuals who smoke. These findings not only emphasize the need for comprehensive smoking cessation programs but also underscore the importance of tailored interventions for individuals with epilepsy, ultimately contributing to improved public health outcomes.

## Data Availability

The original contributions presented in the study are included in the article/Supplementary material, further inquiries can be directed to the corresponding author.
